# A Retained Foreign Body as a Rare Cause of Small Bowel Obstruction (Gossypiboma): A Case Report

**DOI:** 10.7759/cureus.37185

**Published:** 2023-04-05

**Authors:** Arisha Ahmed, Sushil Lohiya

**Affiliations:** 1 Surgery, Krishna Institute of Medical Sciences (KIMS) - Kingsway Hospitals, Nagpur, IND

**Keywords:** foreign body retrieval, pregnancy complications, postoperative complications, laparoscopy, foreign bodies

## Abstract

A retained foreign body (RFB) is a rare but possible complication of surgery. Among the most common retained foreign bodies are sponges, which may include lap pads and gauze pieces. Surgical never events are errors in medical care that are identifiable and preventable but have serious consequences for the patient, making it an important problem in terms of the safety and credibility of a healthcare facility. They also pose a major medicolegal threat to healthcare organizations and a diagnostic challenge for surgeons. Herein, we present the case of a 35-year-old woman who presented with signs and symptoms of acute intestinal obstruction. She revealed a history of Caesarean section 11 months prior. She had a stormy postoperative course then and had to undergo a diagnostic laparoscopy for pus aspiration three months after surgery, where no finding other than pus was reported. Upon presentation at our tertiary care center, she was examined and found to have an RFB for 11 months. She was managed surgically with successful laparoscopic removal of the gossypiboma and consequent resolution of all her symptoms. Though rare, the possibility of an RFB, especially after open surgery, should be kept in mind when diagnosing patients who present with pain, mass in the abdomen, or symptoms of an infection. Laparotomy is the mainstay of treatment for gossypiboma, but successful laparoscopic removal of the RFB provides a definite treatment with the super-added benefits of laparoscopy. Laparoscopic removal of gossypiboma has been reported in the literature and demonstrated in our tertiary care center.

## Introduction

Gossypiboma, textiloma, or cottonoid are the common terms used to describe a mass in the body caused by a retained surgical sponge surrounded by a foreign body reaction. A surgical sponge is the most common type of retained foreign body (RFB) [1]. The exact incidence rate of gossypiboma in clinical practice is difficult to estimate, as cases are underreported. In the abdomen, a retained sponge can invaginate a hollow viscus or cause a mass effect due to fibrous encapsulation. It can go undetected for years if asymptomatic, and when it becomes symptomatic, it causes a great deal of physical agony and mental stress for the patient and monetary compensation, embarrassment, or even loss of job for the surgeon.

We present the case of a 35-year-old female patient who presented with signs and symptoms of acute intestinal obstruction. She revealed a history of Caesarean section 11 months prior and had been more or less asymptomatic since then.

## Case presentation

A 35-year-old woman presented to the gastroenterology outpatient department with complaints of pain in her abdomen and inability to pass stools for five to six days. The pain was dominantly in the infraumbilical region, acute in onset, dull in nature, nonradiating, not associated with food intake or movement, and nonresponsive to oral pain medications. Occasionally, she also felt a slight discomfort in the periumbilical region. She denied any episodes of vomiting or fever and mentioned that she was able to pass flatus.

In early 2021, the patient underwent a laparoscopic myomectomy for a large uterine fibroid. Following that, she received a trial of in vitro fertilization, which resulted in the implantation of three fetuses, of which two were in the uterus and one was tubal ectopic, for which she underwent a second laparoscopy. She carried the uterine twin pregnancy to term and delivered via Caesarean section in January 2022. Her postoperative course was complicated by abdominal pain and nonpassage of flatus, but she was managed conservatively for subacute intestinal obstruction.

In March 2022, she experienced vague abdominal pain and underwent ultrasonography. Free fluid was observed in the pelvis; therefore, the patient underwent a diagnostic laparoscopy, during which pus was aspirated from the pelvis, with no other significant findings.

The patient presented to our tertiary care center with the aforementioned complaints. On examination, she was tachycardic with a heart rate of 105 beats per minute, and the rest of her general examination results were unremarkable. Her abdomen was nondistended and soft but tender in the left iliac fossa and infraumbilical region. A nasogastric tube was inserted, and the patient kept nil per oral and was managed in the ward with fluids and adequate analgesia.

Investigations

**Figure 1:**
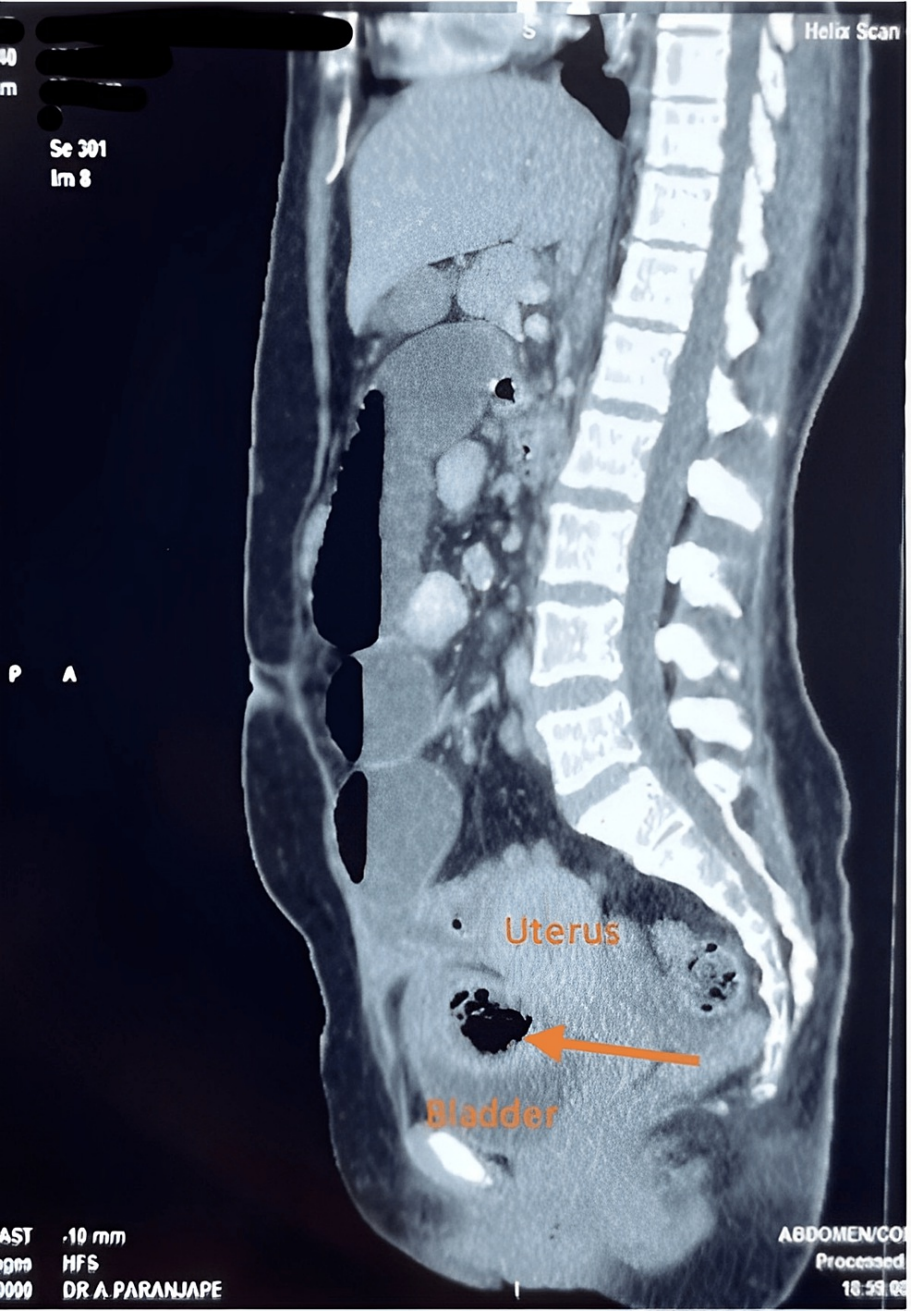
CECT of the abdomen and pelvis showing an oblong low-density structure (orange arrow) encapsulated between the bladder and the uterus, adhering to the bowel. CECT, contrast-enhanced computed tomography

None of the hematological investigations revealed any abnormalities. Erect radiography of the abdomen revealed air-fluid levels. An ultrasonography study was reported to show multiple dilated jejunal loops and an echogenic foreign body of approximately 8 cm, with posterior acoustic shadowing in the lower midline above the bladder. The findings from a contrast-enhanced computed tomography (CT) scan of the abdomen and pelvis resonated with the ultrasonography findings, showing 40-mm wide jejunal loops and jejunoileal junction narrowing. Radiologists also reported a 10 cm × 5 cm oblong low-density structure with thick walls closely adherent to the bowel loops and adjacent uterus (Figure 1).

**Figure 2:**
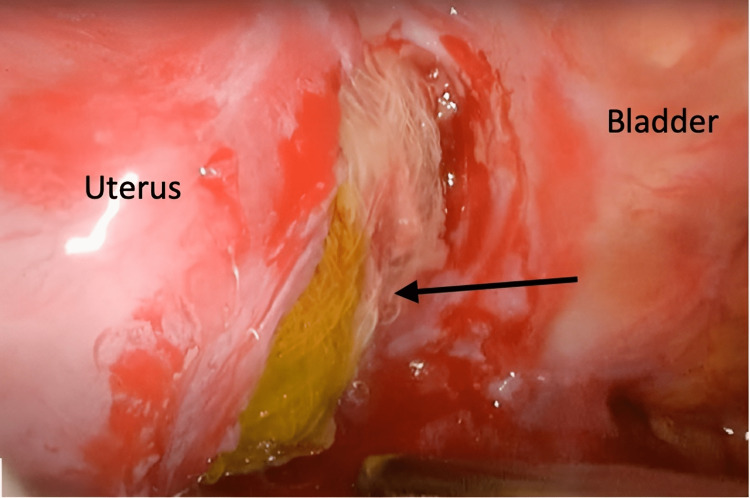
Intraoperative image of the vesicouterine pouch. The laparoscopic image showing a stool-stained mop (black arrow) between the uterus and the bladder on hydrodissection.

Performing a diagnostic laparoscopy with removal of the foreign body with resection and anastomosis of any damaged bowel was decided. All four quadrants of the abdomen were examined. The right and left upper quadrants and the right lower quadrant were normal. In the left lower quadrant, a firm lump was found. The vesicouterine pouch was opened via a hydrodissection, exposing a cotton gauze measuring 10 cm × 15 cm, encapsulated by fibrous tissue, stained with stool, and surrounded by pus (Figure 2).

**Figure 3:**
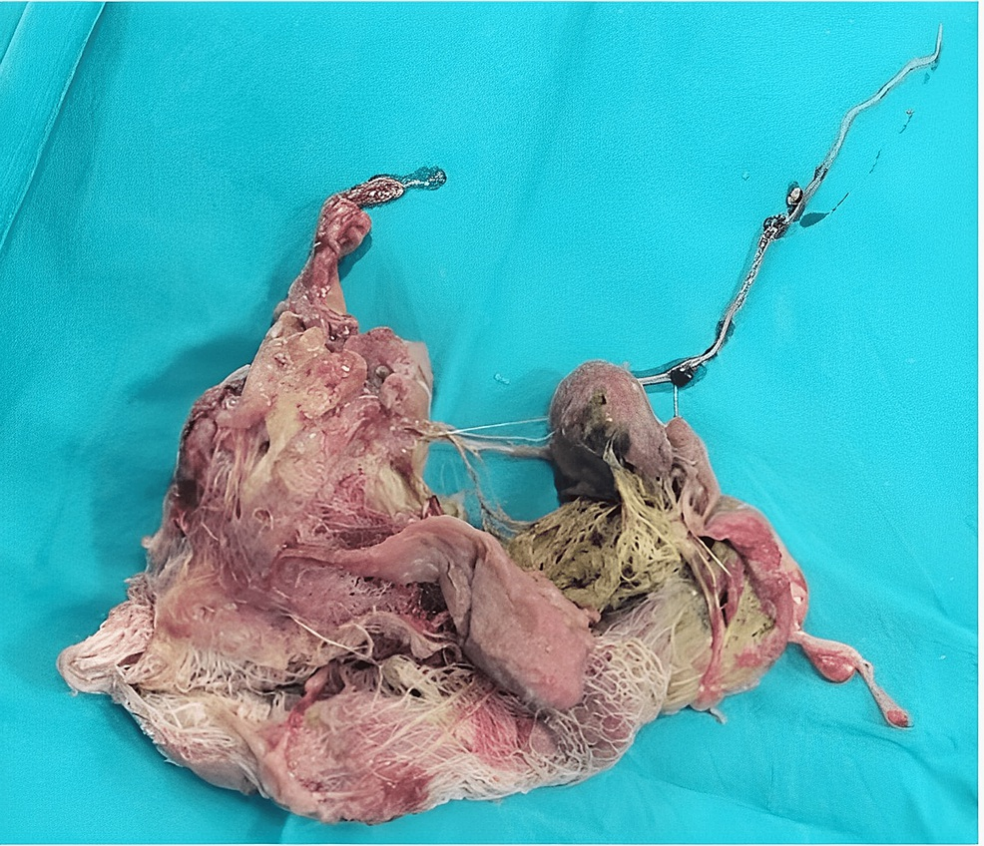
Retained foreign body. Removed the mop from the body.

Along with this lump was an adhered loop of jejunum, which the gauze had invaginated into, causing a perforation. The foreign body was removed laparoscopically, and the perforated bowel loop was resected out. Eight centimeters of distal jejunum was resected, and a side-to-side jejunoileal anastomosis was performed. The retained gauze, about 10 cm × 6 cm, after extraction is shown in Figure 3.

The patient had a difficult postoperative course, with delayed ileus and postoperative pain. The nasogastric tube was removed on postoperative day 2, when the patient could pass flatus and tolerate liquids orally. However, reinsertion was performed on postoperative day 4 because of abdominal distention and nonpassage of flatus. The patient stayed in the hospital for 17 days postoperatively and developed a small local area of collection at the port site in the right iliac fossa, where the RFB was removed from. She was managed for the symptoms of the complication and sent home on postoperative day 17. At the 30-day follow-up, she was in good health and reported tolerating a soft diet well with no further abdominal complaints.

## Discussion

Gossypiboma is derived from the Latin word Gossypium (meaning cotton) and the Swahili word boma (meaning place of concealment). An RFB discovered by a fresh set of surgeons poses a professional and personal dilemma. For this reason, gossypiboma cases remain grossly underreported. However, the incidence of gossypiboma was reported to be 1 in 100-5,000 surgical procedures and one in 1,000-1,500 intra-abdominal operations [2]. Gawande et al. identified three risk factors of gossypiboma: emergency procedures, an unplanned change during the procedure, and the patient's body mass index [3]. Gossypiboma is also more common in female patients who have undergone gynecological procedures owing to the discrepancy between the size of the incision and the area explored [4], which is consistent with our case, where the patient had a lower segment Caesarean section 11 months before the presentation.

An RFB can lie dormant in the body for long periods, and the body deals with it in two ways: first, through an exudative, infectious response that leads to abscesses and fistulas, and second, an aseptic fibrinous response that leads to encapsulation and subsequent mass effect [5,6]. A patient with gossypiboma can present with fever, vomiting, obstruction, intra-abdominal sepsis, fistula formation, and gastrointestinal bleeding. In our case, a laparoscopy performed three months after a Caesarean section revealed a purulent collection in the pelvis, but the sponge was most probably missed because of its location, which was deep in the vesicouterine pouch, and it was covered with adhesions. A retained sponge can also migrate into an adjacent hollow viscus and lead to perforations, fistulae, or obstruction. The most common site for invagination is the small intestine because of its large surface area and thin wall [6]. This is consistent with the finding of jejunal perforation in our case.

A gossypiboma can be difficult to diagnose. Theoretically, all radiological investigations such as ultrasonography, radiography, CT, and MRI can identify RFBs but may be challenging. In the case of radiography, the sponge may be hidden, like in our case, where it was in a pelvic pouch, or the skiagram may be complicated by gas shadows or incidental findings such as renal stones. Our patient's radiograph only showed features of small bowel obstruction. The RFB may not be detected on MRI because the radiopaque marker is not magnetic. For all these cases, a CT examination is the investigation of choice. The characteristic appearance is that of a spongiform pattern with gas bubbles [2].

When a gossypiboma is found, surgical removal of the foreign body is mandatory. The two available options for dealing with a gossypiboma are exploratory laparotomy and diagnostic laparoscopy, both followed by the removal of the RFB and needful surgery. While an open method can be quicker and easier and provide better exposure, laparoscopy has advantages, including a less painful postoperative course, smaller scars, early ambulation, early oral intake, lesser risk of hernia, and overall quicker recovery and return to daily life activities [5,7]. However, laparoscopy may involve a longer operating time because of adhesiolysis and the surgical technique and poses a risk of bowel injury during entry to the abdomen. Reports on laparoscopy used for the retrieval of an RFB are limited, but in appropriately selected patients, it can provide faster recovery and decrease the anxiety of undergoing an operation due to a previous poor experience.

A gossypiboma is classified as a surgical never event. According to the definition of the US National Quality Forum, *never events* are errors in medical care that are identifiable, preventable, and serious in their consequences for patients, making it an important problem in terms of the safety and credibility of a healthcare facility [8]. To prevent this event, various guidelines are followed, such as the frequency of performing the sponge count and the World Health Organization surgical safety checklist. The decision to use and update the safety checklists regularly depends upon the institution and its resources, but the recommendation is to use and update them frequently.

## Conclusions

Though rare, an RFB should not be completely ruled out when making a differential diagnosis. To avoid RFBs, multiple checks at multiple levels should be ensured and the nursing staff should be given proper education about safety checklists and the importance of instrument and gauze counts. Although laparotomy is the go-to procedure for RFB removal, successful laparoscopic removal allows for diagnostic exploration, with the added advantages of early ambulation, decreased postoperative pain, early diet tolerance, and overall early return to normal life.

## References

[1] Aminian A (2008). Gossypiboma: a case report. Cases J.

[2] Kumar P, Shukla P, Tiwary SK, Verma A, Khanna AK (2021). Gossypiboma: an avoidable but not a rare complication. Proc Singap Health.

[3] Gawande AA, Studdert DM, Orav EJ, Brennan TA, Zinner MJ (2003). Risk factors for retained instruments and sponges after surgery. N Engl J Med.

[4] Sankpal J, Tayade M, Rathore J (2020). Oh, my gauze !!! A rare case report of laparoscopic removal of an incidentally discovered gossypiboma during laparoscopic cholecystectomy. Int J Surg Case Rep.

[5] Khanduri A, Gupta J, Ammar H, Gupta R (2022). Laparoscopic removal of retained surgical sponge after caesarean section: a case report. Cureus.

[6] Guţu S, Ţugui I, Guzun V, Cerbadji A, Guţu E, Rojnoveanu G (2022). Gossypiboma as a rare cause of small bowel obstruction: a case report. Chirurgia (Bucur.

[7] Soori M, Shadidi-Asil R, Kialashaki M, Zamani A, Ebrahimian M (2022). Successful laparoscopic removal of gossypiboma: a case report. Int J Surg Case Rep.

[8] Kumar J, Raina R (2017). 'Never events in surgery’: mere error or an avoidable disaster. Indian J Surg.

